# WHAT SAVES A STRESSFUL DAY: PROTECTIVE ROLE OF POSITIVE AFFECTS AGAINST DAILY STRESS

**DOI:** 10.1093/geroni/igac059.1025

**Published:** 2022-12-20

**Authors:** Joanna Hong, Jody Greaney, Jacqueline Mogle, David Almeida

**Affiliations:** The Pennsylvania State University, State College, Pennsylvania, United States; The University of Texas of Arlington, Arlington, Texas, United States; The Pennsylvania State University, University Park, Pennsylvania, United States; The Pennsylvania State University, University Park, Pennsylvania, United States

## Abstract

Experiencing a greater number of stressful days is associated with a heightened risk of cardiovascular disease. This study examined whether positive affect reactivity, the trait-like change in positive affect in response to daily stressors, moderates the association between the number of stressful days and blood pressure. Participants were 664 adults from the National Study of Daily Experiences II (Midlife in the United States sub-study). Participants reported stressors and positive affect across 8 days and provided resting blood pressure measures during a separate clinic visit. A greater number of stressful days was associated with increased systolic blood pressure, but only among individuals with higher positive affect reactivity (B = 15.11, SE = 6.36, p = 0.02). Results suggests that individuals who maintain positive affect when experiencing stressors may have lower risk of heightened systolic blood pressure, contributing to the growing evidence that positive affective reactivity may be protective against daily stress.

